# Experimental infection of cows with newly isolated Akabane virus strain (AKAV-7) causing encephalomyelitis

**DOI:** 10.1186/s13567-016-0349-6

**Published:** 2016-06-10

**Authors:** Hyeyeoun Lee, Hansol Jeong, Surim Park, Myeon-Sik Yang, Jongwon Kim, Jaehyun Bae, Yonghwan Kwon, Min-Su Kim, Jae-Ku Oem, Myoung-Heon Lee, Chae-Woong Lim, Bumseok Kim

**Affiliations:** College of Veterinary Medicine, Chonbuk National University, Iksan, 54596 Korea; National Institute of Environmental Research, Ministry of environment, Incheon, 22689 Korea; Division of Animal Diseases Diagnosis, Animal and Plant Quarantine Agency, Gimcheon, 39660 Korea

## Abstract

Akabane virus (AKAV), an arthropod-transmitted bunyavirus, is a major cause of congenital abnormalities and encephalomyelitis in ruminants. In 2010, there was a major outbreak of encephalomyelitis in Korea and fifteen AKAV strains, including AKAV-7, were isolated from cows. To identify the neuropathogenicity of AKAV-7, we performed experimental infection of cows. Six-month-old female Korean Holstein dairy cattle were inoculated with AKAV-7 by various routes, including intracerebral (IC), intrasubarachnoid space (IS), subcutaneous (SC) and intravenous (IV); a separate group was vaccinated before intravenous infection. Five of the six cows in the IC group and two of the six cows in the IS group showed clinical signs such as locomotor ataxia and paralysis of the hind limbs. Three of six cows died after IC infection 9–12 days post infection (dpi). Histopathologic changes such as nonsuppurative encephalomyelitis were confirmed in various parts of the central nervous system in the IC, IS and SC groups. Early onset of neutralizing antibodies in the serum and lower viral mRNA levels in the peripheral blood mononuclear cells (PBMCs) and various tissues in the vaccinated group was noticeable compared to the unvaccinated group (IV group). We suggest that the AKAV vaccine currently used in Korea may be partially effective for protection against AKAV-7 in cows.

## Introduction

Akabane virus (AKAV), a member of the *Orthobunyavirus* genus in the family *Bunyaviridae*, is transmitted by an arthropod vector such as *Culicoides* biting midges. This arthropod-borne virus (arbovirus) circulates widely in the tropical and temperate zones of the world [1–4]. It is distributed throughout Southeast Asia, East Asia, Australia and the Middle East [5]. Akabane disease is an AKAV related disease of ruminants causing congenital abnormalities including abortions, premature births, stillbirths and congenital malformations such as arthrogryposis-hydranencephaly syndrome [6].

Similar to other members of the genus *Orthobunyavirus*, AKAV has a lipid envelope and a genome that forms three segments of single-stranded negative-sense RNA, referred to as S (small), M (medium) and L (large) depending on their size [7]. With phylogenetic analysis, AKAV can be classified into four distinct genogroups (I–IV) and genogroup I is subdivided into two subgroups (Ia and Ib) [6, 8, 9].

In Japan, the prototype AKAV strain was first isolated from mosquitoes in 1959 [10]. Sporadic outbreaks of Akabane disease have been reported in many other countries [11–13]. In Korea, the first outbreak of Akabane disease was reported in 1980 [14]. The main clinical signs of Akabane disease are congenital abnormalities in the fetus; however, the AKAV variant strain Iriki was isolated from 10 calves in Japan with neurological signs and nonsuppurative encephalitis in 1984 and caused encephalitis in calves by experimental inoculation [15]. Since then, additional AKAV strains also caused encephalomyelitis of calves and adult cows in Taiwan [4]. In 2000, encephalomyelitis associated with AKAV infection in cows was also reported in Korea [16].

From late summer to late autumn 2010, a major outbreak of Akabane viral encephalomyelitis in adult cows occurred in the southern part of Korea. More than 500 cattle showed primarily neurological signs such as locomotor ataxia, astasia, tremors, hypersensitivity and paralysis of the fore- or hind limbs. Nonsuppurative encephalomyelitis manifesting mainly as lymphohistiocytic perivascular cuffing, gliosis and neuronal degeneration was observed in the brain and spinal cord of infected cows on histopathologic examination [17]. The fifteen strains of AKAV containing AKAV-7/SKR/2010 (AKAV-7) isolated from the affected cows were classified into genogroup Ia within the Iriki strain [18]. In previous studies, considerable diversity in the antigenic properties and virulence was observed among field isolates of AKAV [15, 19–21]. However, little has been investigated regarding the neuropathogenicity of AKAV strains causing nonsuppurative encephalomyelitis in cows. Moreover, AKAV genogroup II strains are being used as vaccines in Korea [18]. Hence an efficacy assessment of a current vaccine for the newly isolated AKAV is necessary. Therefore the aim of this study was to verify the neuropathogenicity of AKAV-7 causing encephalomyelitis by experimental infection in cows through various inoculation routes.

## Materials and methods

### Animals

Six-month-old female Korean Holstein dairy cattle were used for this experiment. The AKAV antibody titers of all cows were seronegative by the virus neutralization test (VNT) before experimental procedures. All cows were clinically healthy and kept in the animal facility at a college of veterinary medicine, Chonbuk National University, under standard conditions prescribed by the Institutional Guidelines. This experiment was approved by the Institutional Animal Care and Use Committee (IACUC) of Chonbuk National University.

### Virus

The twice-passaged AKAV-7 was provided by the Viral Disease Diagnostic Laboratory of Animal Disease Diagnostic Division, Animal and Plant Quarantine Agency, Republic of Korea. BHK-21 cells were used to grow the virus. After removal of cell debris by 15 min of ultracentrifugation at 9000 rpm, the 250 mL culture medium, including the virus, was concentrated to a 10 mL final volume by Vivacell 250 (Sartorius Stedim Biotech, Goettingen, Germany) to increase the virus titer. Virus titer was referred to as the tissue culture infective dose (TCID_50_) and was based on endpoint titration assay. The final virus titer was 10^6.866^ TCID_50_/mL. All protocols were performed at 4 °C.

### Virus inoculation procedure and sample collection

Animals were divided into intracerebral (IC), intrasubarachnoid (IS), subcutaneous (SC), intravenous (IV), vaccinated before intravenous inoculation, and negative control groups. A mixture of tiletamine/zolazepam (80 mg/kg of body weight, Zoletil; Virbac SA, Carros, France) and xylazine (50 mg/kg of body weight, Rompun; Bayer Healthcare, Seoul, Korea) was used to anesthetize cows by intramuscular injection. For IC inoculation, the right side of the skull was trepanned to a depth of approximately 5 mm. One milliliter inoculum was injected into the frontal lobe. For IS inoculation, 5 mL inoculum was inoculated into the subarachnoid space at the level of the thoracolumbar part. The SC group was inoculated with 5 mL of inoculum subcutaneously through the skin near the back of the neck. For the IV group, 5 mL inoculum was injected through the jugular vein. In the vaccinated group, cows were vaccinated with a live vaccine (Akabane Cattle Vac, Daesung, Korea) intramuscularly and inoculated intravenously with 5 mL inoculum 3 weeks after vaccination. After infection, cows were observed daily to monitor clinical condition. Blood samples were collected every 2 days after inoculation to check for viremia and serology. Because peripheral blood mononuclear cells (PBMCs) having viral antigen can possibly cross blood–brain barrier and induce encephalitis, PBMCs were isolated by Histopaque-1077 (Sigma, Dorset, UK) and used for viral antigen detection using RT-PCR. The cows were sacrificed under deep anesthesia 7 days post-infection (dpi) in the IC and IS group and 21 dpi in the IS, SC, IV and vaccinated groups. After sacrifice, tissue samples from the brain, spinal cord, spleen and lymph nodes were collected.

### Viral neutralizing antibody test (VNT)

The collected sera were first heat-inactivated at 56 °C for 30 min. MDBK cells were raised in alpha-minimum essential medium (α-MEM; HyClone, Logan, USA) containing 5% fetal bovine serum and antimycotic antibiotics (Welgene, Daegu, Korea). Flat-bottomed 96-well tissue culture plates were used. Briefly, two-fold serial dilutions of the serum sample (50 μL) were mixed with equal volume of virus containing 200 TCID_50_/0.1 mL and inoculated with MDBK cells. Microscopic examination was conducted on the plates after 3 or 5 days for evidence of virus-specific cytopathic effects (CPE). Antibody titers were expressed as the reciprocal of the highest serum dilution at which the CPE were inhibited. A serum dilution titer of 2 (log_2_) or greater was considered seropositive.

### Competitive enzyme-linked immunosorbent assay (C-ELISA)

The AKAV-specific antibodies in the serum were detected using the ID Screen^®^ Akabane C-ELISA kit (IDvet Innovative Diagnostics, Montpellier, France), as recommended by the manufacturer. The inhibition of monoclonal antibody binding after incubation of the antigen with each test serum was calculated as the percent inhibition of the monoclonal antibody with respect to the reference negative control serum by using the following formula: percent inhibition (PI) = 100 − [(test serum OD)/(mean negative control OD) × 100]. The test serum sample was considered to be antibody-positive if the PI was ≥70%.

### Quantitative real-time polymerase chain reaction (real-time PCR)

Total viral RNA was isolated from PBMCs and tissue using the Easy-Spin Total RNA extraction kit (GeneAll, Seoul, Korea). Following incubation with RNase-free DNase I (Promega, Madison, WI), reverse transcription was performed using a random primer and MultiScribeTM MuLV reverse transcriptase (Thermo Fisher Scientific, Waltham, MA, USA) following the manufacturer’s instructions. cDNA was subjected to real-time PCR on a CFX96 Real-Time PCR Detection SystemTM (Bio-Rad Laboratories, Hercules, CA, USA) using SYBR Green I dye. PCR amplifications were performed with primers in a total volume of 15 μL containing 0.3 μL of each primer (Table 1), 7.5 μL of SYBR Green I dye (Takara Bio Inc.), and nuclease-free PCR-grade water after initial denaturation at 95 °C for 10 min and 50 cycles (95 °C for 10 min and 60.5 °C for 30 min). After the reaction was completed, specificity was verified by melting curve analysis. Quantification was confirmed by comparing Ct values of each sample with normalization to glyceraldehyde-3-phosphate dehydrogenase (GAPDH).

**Table 1:** **Primer sequences of real-time PCR**

RNA target	Primer sequences	Product size
Akabane virus S RNA	F: 5′-GCTAGAGTCTTCTTCCTCAACCAGAA-3′ R: 5′-AAAAGTAAGATCGACACTTGGTTGTG-3′	26 bp 26 bp
Cow GAPDH	F: 5′- GATGGTGAAGGTCGGAGTGAAC-3′ R: 5′- GTCATTGATGGCGACGATGT-3′	22 bp 20 bp

### Histopathologic examination

The tissues were fixed in 10% phosphate-buffered formalin, routinely processed and then embedded in paraffin. Tissue Sections (6 μm) were prepared using a microtome (HM-340E, Thermo Fisher Scientific, MA, USA), placed on a glass slide and stained with haematoxylin and eosin (H&E). H&E staining was performed following standard procedures. With 6 μm-thick paraffin sections, histopathological examination in the frontal, parietal and occipital lobes of the cerebrum, the cerebellum, brain stem (mid brain, pons and medulla oblongata) and spinal cord (cervical, thoracic and lumbar) was performed.

### Immunohistochemical evaluation

For immunohistochemical staining, the prepared 6 μm-thick tissue on glass slides was deparaffinized, rehydrated, and then immersed in an antigen retrieval solution (Dako, Seoul, Korea) for 30 min at 100 °C. A 3% peroxidase solution followed by 10% normal goat serum was used for blocking. The ImmPACT DAB peroxidase substrate kit (Vector Laboratories, Burlingame, CA, USA) was used for immunostaining. Rabbit antiserum against the 93FMX strain isolated in Korea (reference strain in Korea) was used as the primary antibody. The antiserum was diluted 1 in 1000 and was incubated with the tissue sections for 3 h at room temperature. The anti-mouse/rabbit immunoglobulin G (ImmPress Universal Reagent, Vector Laboratories, Burlingame, CA, USA) was used as the secondary antibody and peroxidase substrate solution was used until desired stain intensity develops. Finally, the sections were counterstained with hematoxylin and processed for mounting with mounting medium.

### Statistical analysis

Some data are expressed as mean ± SEM. Differences between the two groups were compared using a two-tailed Student’s *t* test. A value of *p* < 0.05 was considered a statistically significant difference between groups.

## Results

### Mortality and clinical features of cows

After AKAV-7 injection, all cows were daily observed to identify clinical signs. Five of the six cows with neurologic signs such as ataxia, astasia and paralysis of the fore- and hind limbs were from the IC inoculation group, with symptoms starting 5 and 6 dpi. Two of three cows died at 9 dpi, and one of three cows died at 12 dpi after IC inoculation. After IS injection, two of six cows showed astasia, ataxia, and paralysis of the hind limbs; however, these cows survived until sacrifice at 7 and 21 dpi, respectively. In the IV and SC injection groups, there were no clinical signs during the experimental periods.

### Serological surveys by VNT and C-ELISA

**Figure 1:**
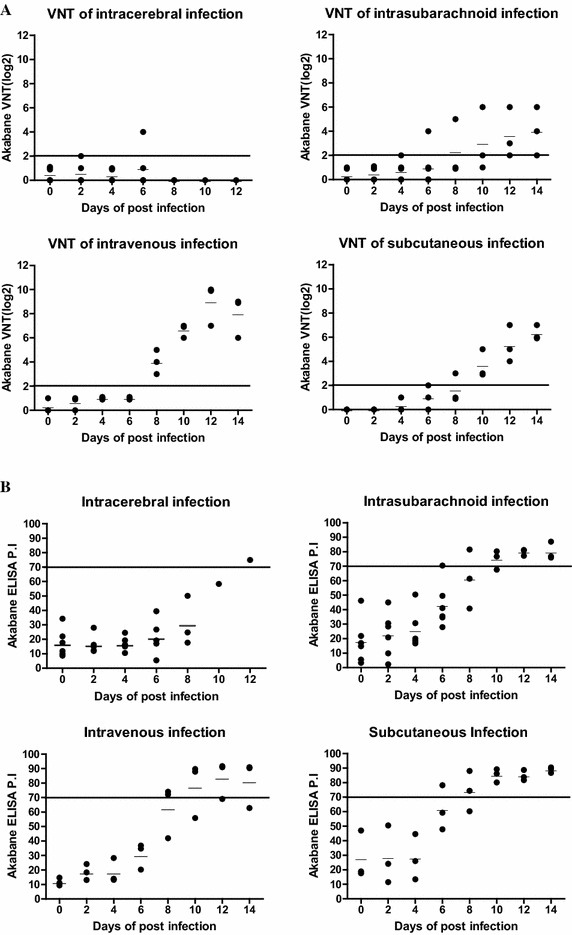
**Detection of neutralizing antibodies and specific antibodies against AKAV in the serum of inoculated cows using VNT and C-ELISA.** **A** In VNT, animals were considered seropositive when the neutralizing antibody titer (log_2_) rose above a threshold of 2 (reference line). **B** In C-ELISA results, P.I. values greater than 70% were considered seropositive (reference line).

As shown in Figure 1A, the VNT titers (log_2_) of only two serum samples from one cow were positive at 2 and 6 dpi in the IC group. In contrast, 11 of 36 serum samples from the IS inoculation group were seropositive. The titers started to increase at 4 dpi and steadily maintained in the range of 2–6 (log_2_) at 14 dpi. At 12 dpi, all cows in the IS group had positive neutralizing antibodies. After IV injection, all cows were seropositive at 8 dpi. The titers (log_2_) of the IV group started to increase at 8 dpi and appeared in the range of 6–9 at 14 dpi. The highest titer in the IV group was 10 at 12 dpi. In the SC inoculation group, all cows had positive neutralizing antibodies at 10 dpi. The titers (log_2_) were 2 at 6 and 8 dpi and ranged from 6–7 at 14 dpi. The highest titer in the SC group was 7 at 12 and 14 dpi.

In the C-ELISA test, only one cow in the IC group had a seropositive titer (>70 titer, PI) 12 dpi because most cows died 9 dpi. After IS inoculation, one of six cows appeared seropositive 6 dpi and all cows in the 21 dpi group were seropositive 12 dpi. In the IV group, two of three cows started to show seropositivity 8 dpi and the mean ELISA titer rose to 81.4% at 14 dpi. The highest titer in the IV group was 91.7% at 12 dpi. After SC inoculation, one of three cows was seropositive 6 dpi and all cows had seropositive titers from 10 dpi. The titers increased steadily until 14 dpi, and the highest titer was 90.7% at 14 dpi in the SC group (Figure 1B).

Results of serum surveys revealed a positive correlation between VNT and C-ELISA in the IC, IS, IV and SC injection groups. The correlation rates for VNT and C-ELISA were 80.6% (25/31) in the IC group, 91.6% (33/36) in the IS group, 83.3% (20/24) in the IV group and 95.8% (23/24) in the SC group, respectively (data not shown). All antibody results indicated that antibody production levels were lower in the IC group compared to others.

### Viremia and distribution of AKAV antigens in various tissues

**Figure 2:**
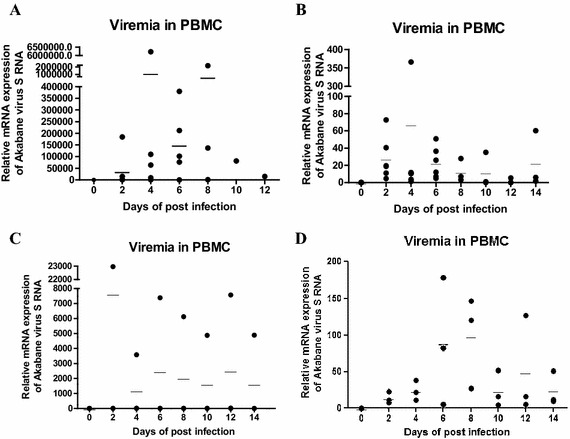
**Detection of AKAV antigen in PBMCs using quantitative real-time PCR to identify viremia.** **A** Results of relative quantification in real-time PCR for detecting AKAV antigen in PBMCs from six cows of the IC group. In the 21 dpi planned group, two of three cows died 9 dpi and one of three cows died 12 dpi. **B** Results from six cows of the IS group, **C** from three cows of the IV group and **D** from three cows of the SC group.

After IC injection, two viral load peaks were observed in PBMCs 4 and 8 dpi. Viremia was observed as a peak after IS injection 4 dpi and decreased after 6 dpi. After IV injection, peak viremia was seen in one cow 2 dpi. Viral antigen in PBMCs started to decline after 4 dpi. Two viremia peaks were detected in PBMCs 6 or 8 dpi and 12 dpi after SC injection (Figure 2).

**Figure 3:**
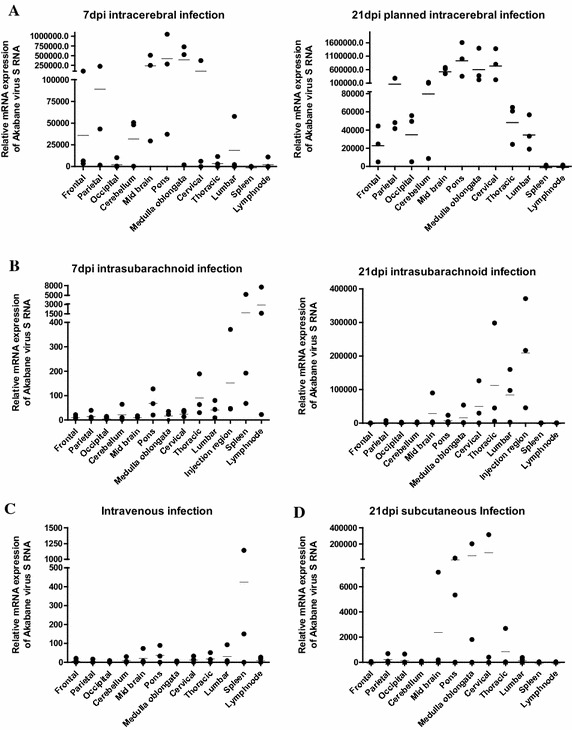
**Detection of AKAV antigen in the cerebrum, cerebellum, brain stem, spinal cord and organs of the immune system using quantitative real-time PCR.** **A** Results of relative quantification in real-time PCR for detecting AKAV antigen in various tissues from three cows of the IC group 7 and 21 dpi respectively, **B** from three cows of the IS group 7 and 21 dpi respectively, **C** from three cows of the IV group 21 dpi and **D** from three cows of the SC group 21 dpi.

After IC inoculation, viral RNA was primarily detected in the parietal cerebrum, brain stem and cervical spinal cord 7 and 21 dpi. In the IS group, at 7 dpi, viral RNA was measured mostly in the spleen, lymph nodes and injection region (thoracolumbar part). Similar results were found 21 dpi in the IS group, except in the organs of the immune system. In the IV group, the detection of viral antigen was limited to the spleen. Viral antigen was observed mildly or not determined in other tissues. Viral RNA was identified markedly in the brain stem and cervical spinal cord after SC inoculation (Figure 3).

### Histopathological lesions and immunohistochemical characteristics for AKAV antigens in cows

Histopathologically, nonsuppurative encephalomyelitis was characterized by perivascular cuffing (PVC) of lymphocytes and macrophages, meningitis with numerous lymphocytes and glial nodules consisting of microglial cells (gliosis) in the central nervous system (CNS).

**Figure 4:**
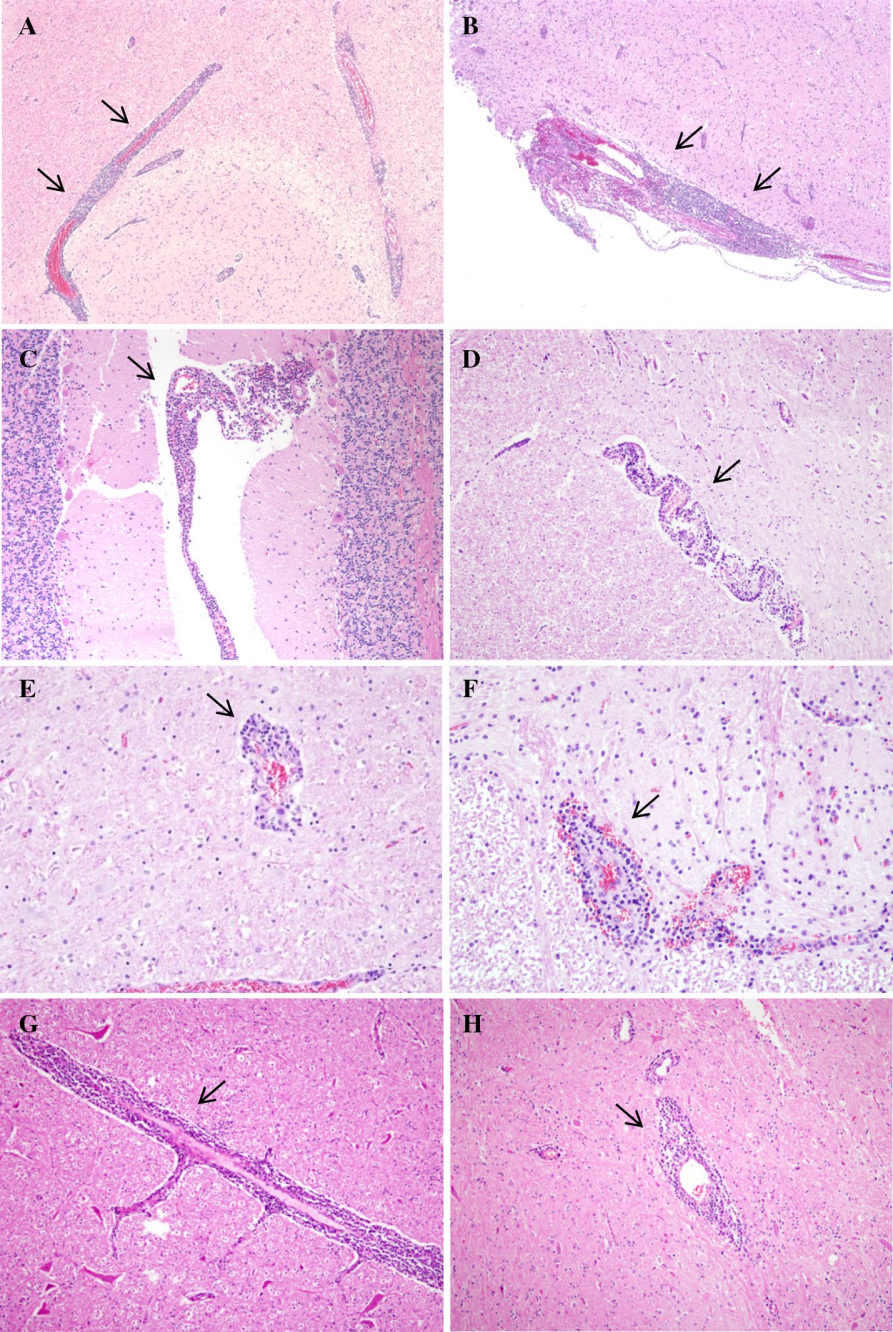
**Nonsuppurative encephalomyelitis in central nervous tissues of the IC, IS and SC groups with AKAV-7.** **A** PVC (arrows) in the frontal lobe of the IC group 7 dpi, H&E. Original magnification, ×40. **B** Meningitis (arrows) in the frontal lobe of the IC group 7 dpi, H&E. Original magnification, ×40. **C** Meningitis (arrow) in the cerebellum of the IC group 7 dpi, H&E. Original magnification, ×40. **D** PVC (arrow) in the cervical spinal cord of the IC group 7 dpi, H&E. Original magnification, ×100. **E** PVC (arrow) in the mid brain of the IS group 21 dpi, H&E. Original magnification, ×200. **F** PVC (arrow) in the lumbar spinal cord of the IS group 21 dpi, H&E. Original magnification, ×200. **G** PVC (arrow) in the medulla oblongata of the SC group 21 dpi, H&E. Original magnification, ×100. **H** PVC (arrow) in the cervical spinal cord of the SC group 21 dpi, H&E. Original magnification, ×100.

After IC inoculation, nonsuppurative encephalomyelitis was observed in the cerebrum from all cows at 7 dpi and one of three cows in the 21 dpi planned group (Figures 4A and B). Two of three cows had pathological lesions in the cerebellum 7 dpi. In the brain stem, pathological lesions were identified from two of three cows 7 dpi and two of three cows in the 21 dpi planned group. One of three cows showed PVC in the cervical spinal cord 7 dpi (Figure 4D). In the IS group, lesions were observed in the brain stem and lumbar spinal cord from one of three cows 21 dpi (Figures 4E and F). However no pathological lesions in the cerebrum and cerebellum were observed.

After IV inoculation, there were no pathological changes in the CNS. Otherwise, two of three cows in the SC group had encephalomyelitis, mainly in the brain stem and also in the parietal lobe of the cerebrum and cervical spinal cord (Figures 4G and H). A summary of these results is shown in Table 2. None of these changes were shown in the CNS of the control group.

**Table 2:** **Incidence of histopathological lesions in the CNS of each group (** ***n*** **=** **3) infected with AKAV-7**

Different CNS area	IC group		IS group		Unvaccinated IV group	Vaccinated IV group	SC group
	7 dpi	21 dpi^a^	7 dpi	21 dpi	21 dpi	21 dpi	21 dpi
Cerebrum	3/3	1/3	0/3	0/3	0/3	0/3	1/3
Cerebellum	2/3	0/3	0/3	0/3	0/3	0/3	0/3
Brain stem	2/3	2/3	0/3	1/3	0/3	0/3	1/3
Spinal cord	1/3	0/3	0/3	1/3	0/3	0/3	1/3

^a^Two of three cows died 9 dpi and one of three cows died 12 dpi after IC inoculation.

**Figure 5:**
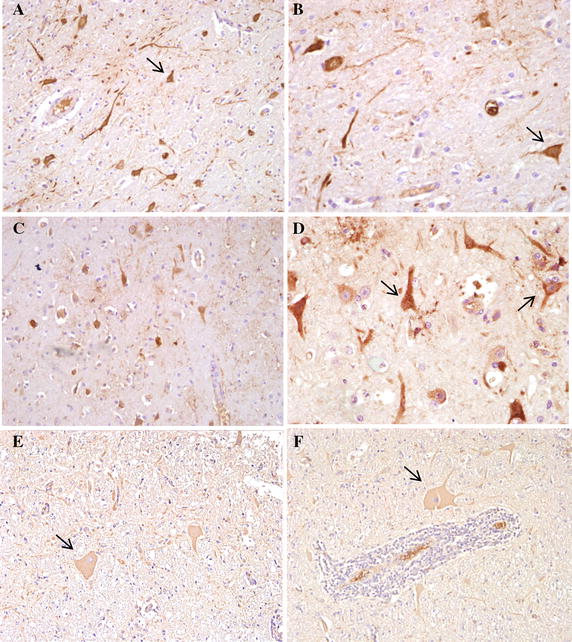
**Immunohistochemical characteristics in the central nervous tissues of cows in the IC and SC groups with AKAV-7.** **A** Detection of AKAV-7 antigens within the cytoplasm of neurons (arrow) and axons in the mid brain of the IC group 7 dpi, IHC. Original magnification, ×200. **B** Detection of AKAV-7 antigens (arrow) in the mid brain of the IC group 7 dpi, IHC. Original magnification, ×400. **C** Detection of AKAV-7 antigens in the frontal lobe of the IC group 21 dpi, IHC. Original magnification, ×200. **D** Detection of AKAV-7 antigens (arrows) in the parietal lobe of the IC group 21 dpi, IHC. Original magnification, ×400. **E** Detection of AKAV-7 antigens within the cytoplasm of neurons (arrow) and axons in the pons of the SC group 21 dpi, IHC. Original magnification, ×200. **F** Detection of AKAV-7 antigens (arrow) in the pons of the SC group 21 dpi, IHC. Original magnification, ×200.

Immunohistochemistry (IHC) was performed to detect AKAV antigens in the neurons and nerve axons. In the IC group, positive viral antigens of the neuron were observed predominantly in the frontal and parietal lobe of the cerebrum and brain stem 7 and 21 dpi (Figure 5). Virus antigens were observed in the pons 21 dpi after SC inoculation (Figures  5E and F).

### Efficacy assessment of AKAV vaccine against AKAV-7 intravenous infection

To evaluate the effect of the AKAV vaccine currently used in Korea on AKAV-7 infection, VNT, C-ELISA and real-time PCR in the unvaccinated group (IV group) and vaccinated group was performed.

**Figure 6:**
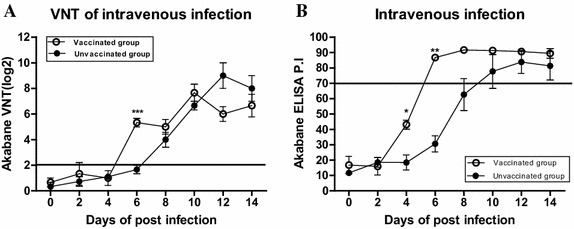
**Efficacy assessment of the AKAV vaccine against AKAV-7 IV infection.** In VNT, animals were considered AKAV-seropositive when the neutralizing antibody titer (log_2_) rose above a threshold of 2 (reference line). In C-ELISA results, P.I. values greater than 70% are considered positive for AKAV antibodies (reference line). Empty dots indicate the vaccinated group before IV infection and filled dots indicate the IV group without vaccination. **A** Neutralizing antibody titers resulting from VNT in the vaccinated group. **B** AKAV-7 antibodies detected by C-ELISA. Data are presented as mean ± SEM per group; *p < 0.05; **p < 0.01; ***p < 0.001.

At 6 dpi, the viral neutralization antibody titers (log_2_) of the vaccinated group were significantly higher than those of the unvaccinated group in VNT (*p* < 0.001). In addition, the mean antibody titer (log_2_) of the unvaccinated group was seronegative [<2 titer, (log_2_)] at 6 dpi (Figure 6A).

In C-ELISA, the mean antibody titers of the vaccinated group were seropositive (>70 titer, PI) 6 dpi. Moreover, the viral antibody titers of the vaccinated group were significantly higher than those of the unvaccinated group 4 dpi (*p* < 0.05) and 6 dpi (*p* < 0.01) (Figure 6B). The viral antibody titers of the unvaccinated group were seronegative in VNT and ELISA until 6 dpi. These results suggest that the antibody titers of the vaccinated group appeared seropositive earlier, and the mean antibody titers were higher 6–10 dpi in VNT and C-ELISA compared to those of the unvaccinated group. The serological studies revealed a positive relationship between the VNT and C-ELISA in the vaccine-treated group, with a coincidence rate of 91.7% (22/24) (data not shown).

Viral antigens in PBMCs from the vaccinated group were detected 4 and 8 dpi; however, compared to the unvaccinated group, the mean viral load in PBMCs was much less during the experiment (Table 3). In both vaccinated and unvaccinated group, viral RNA was low in central nervous tissues (Table 4). In addition, there were no histopathological changes in the CNS of the vaccinated and unvaccinated group.

**Table 3:** **Results of quantitative real-time PCR for the AKAV antigen in PBMCs**

Days post infection	Unvaccinated IV group	Vaccinated IV group
0	0	0
2	76 580.2 ± 7651.9	6.4 ± 2.4
4	1199.9 ± 1191.5	238.5 ± 221.2
6	2464.7 ± 2459.4	133.9 ± 129.8
8	2044.7 ± 2036.4	440.0 ± 433.0
10	1629.5 ± 1622.6	16.4 ± 12.8
12	2530.5 ± 2518.8	36.4 ± 20.2
14	1632.1 ± 1628.6	10.7 ± 4.0

Data are presented as mean ± SEM per group.

**Table 4:** **Results of quantitative real-time PCR for the AKAV antigen in various tissues**

Tissues	Unvaccinated IV group	Vaccinated IV group
Frontal lobe	9.8 ± 6.0	2.4 ± 2.4
Parietal lobe	6.3 ± 5.2	2.2 ± 2.2
Occipital lobe	3.2 ± 2.7	2.4 ± 2.4
Cerebellum	11.8 ± 9.2	2.9 ± 2.9
Mid brain	26.6 ± 23.4	4.2 ± 4.2
Pons	40.6 ± 26.0	4.5 ± 4.5
Medulla oblongata	4.2 ± 2.1	7.5 ± 7.5
Cervical spinal cord	15.5 ± 9.5	9.1 ± 9.1
Thoracic spinal cord	22.1 ± 15.2	7.1 ± 7.1
Lumbar spinal cord	33.8 ± 29.7	3.3 ± 3.3
Spleen	430.6 ± 358.2	2007.0 ± 1968.3
Lymph node	14.7 ± 7.9	1758.9 ± 1750.0

Data are presented as mean ± SEM per group.

## Discussion

There was a previous study on experimental AKAV infection of pregnant cows and calves with genogroup II strains, resulting in congenital abnormalities and nonpurulent encephalomyelitis, respectively [22, 23, 24]. Although some studies regarding AKAV infection were reported, the precise mechanisms of AKAV infection causing neuropathology in animals after birth still remain to be determined. In the present study, we investigated the neuropathogenicity of a newly isolated AKAV-7 from cows by inoculating them via IC, IS, IV and SC routes. To evaluate neurovirulence, neuroinvasiveness and antibody production, several tests such as clinical, histopathological, serological analysis [25] and real-time PCR were performed.

A serosurveillance study in Korea demonstrated that seropositive rates for AKAV were 14.2% in aborted calves before a massive outbreak in 2010 [26]. In 2010, the VNT titers were higher than 4 (log_2_) in most affected cows. Among the tested sera, the highest VNT titers were 12 (log_2_) [27]. Likewise, the VNT titers of all cows in the present study showed seropositivity after 12 dpi except in the IC inoculation group. The highest VNT titers were 10 (log_2_) at 12 dpi in the IV group. In contrast, only two VNT titers and one ELISA titer of samples were seropositive in the IC group. The few seropositive samples from the IC group could be related to severe neurological signs and mortality compared to other groups. We speculate that direct injection of AKAV into the brain can not efficiently stimulate immunity since brain is immune privileged organ and subsequently fails to control virus replication in the brain.

Akabane virus strain distribution in the CNS and PBMCs was detected by real-time PCR. Based on the viral RNA levels in PBMCs, these was a tendency to show two peaks after infection, although all results in the groups varied according to inoculation routes. Even when the virus was inoculated in the frontal lobe of the cerebrum in the IC group, the viral antigens were detected primarily in the brainstem. Interestingly, the results from some cows in the IC group showed that viral spread reached the lumbar spinal cord. In addition, viral RNA in the IS group was mostly detected in the injection region (thoracolumbar region) and organs of the immune system 7 dpi in addition to the cervical spinal cord and mid brain 21 dpi. Several studies have reported viral antigens of the Iriki strain were detected in the brain or/and spinal cord of mice and two calves after IC inoculation [15, 28]. Likewise, we found that viral antigens were distributed widely throughout the CNS of cows after IC and IS inoculation.

The cows infected with AKAV in 2010 showed similar clinical signs to those infected with the Iriki strain, including locomotor ataxia, astasia and hind or forelimb paralysis. Histopathologically, the infected cows showed encephalomyelitis with meningitis, PVC and gliosis [17]. Histopathological examination of the present study confirmed encephalomyelitis in the CNS of cows and the variation of pathogenicity depending on the inoculation route after AKAV-7 infection. In the present study, five of six and two of six cows showed clinical signs after IC and IS injection, respectively. Furthermore, three of six cows in the IC group died before sacrifice. These results are comparable to a previous study that revealed severe encephalitis appeared in calves after IC inoculation with the Iriki strain [15]. Histopathological examination revealed that IC infection is more neurovirulent than other routes and that cows died from nonsuppurative encephalomyelitis after IC inoculation. Several reasons for this could exist. First, the virus was injected directly into the CNS (frontal lobe of cerebrum) of the IC group, leading direct viral infection in the neuron and subsequently causing severe neurological symptom. Second, low titers of neutralizing antibody in the IC group were detected. No marked clinical signs or histopathological changes were observed after IV infection.

As genogroup II strains were primarily isolated from Korea before 2010 [29], these strains were used as vaccine strains. To verify the effectiveness of the current AKAV vaccine for preventing AKAV-7 classified into genogroup Ia, cows were vaccinated 3 weeks before IV infection and compared to the IV group. There were significant differences in antibody titers of VNT 6 dpi and ELISA 4 and 6 dpi between the vaccinated and IV groups. Seroconversion of the vaccinated group occurred earlier, 6 dpi, compared to the IV group. Likewise, faster developing antibody titers could be contributed to protection from AKAV by suppressing virus replication and distribution. Such idea can explain that the viral load of the vaccinated group showed a low level in CNS compared to unvaccinated group. Taken together, these data suggest that the present AKAV vaccine in Korea is likely to be protective against AKAV-7.

To study the neuropathogenicity of AKAV-7, intradermal (ID), IV and SC infection were performed to mimic the infection mechanism of arbovirus. Cows in the ID group (data not shown) showed no significant clinical signs or histopathological changes. This could be because ID inoculation with the exact volume to skin was limited in cows. Although the IV and SC groups showed no obvious clinical signs, severe encephalomyelitis was identified in the SC group on histopathological examination. Furthermore, more viral antigens were detected mostly in the brain stem and cervical spinal cord compared to other organs of SC groups. Therefore, viral encephalomyelitis in the SC group was confirmed by detecting viral antigens and on histopathology even though there were no clinical signs. From this perspective, SC inoculation might be more appropriate for performing experimental AKAV-7 infection in cows than IV inoculation. Although there is no evidence why SC group showed encephalitis, but IV group did not, we speculate that some antigen presenting cells in the subcutaneous area might contribute to replication and delivery of virus into the CNS. Further study will be necessary to elucidate the detailed neuropathogenic mechanism of AKAV-7.

It is important to consider the differences and similarities between infection with artificial routes (the present study) and natural infections. Based on previous study regarding outbreak in Korea in 2010, around 500 cattle with neurological signs in more than 100 farms were reported [17]. Serological investigation of all cattle from the outbreak farms showed that over 70% of the cattle had AKAV specific antibodies. Such information clearly indicates that natural AKAV infection did not always induce encephalomyelitis in all infected cattle presumably by natural barriers and host protective immunity. Sporadic outbreak of encephalomyelitis can explain why not all of the infected cattle with limited numbers via different routes showed clinical signs and neuropathology, and why data variations commonly occur in the same experimental group of our study.

In summary, this study demonstrated the neuropathogenicity of AKAV-7 inoculated via IC, IS and SC routes, which caused encephalomyelitis. Moreover, the current AKAV vaccine in Korea is considered to have partial protective effects against AKAV-7.
